# Deoxycholic acid induces the overexpression of intestinal mucin, MUC2, via NF-kB signaling pathway in human esophageal adenocarcinoma cells

**DOI:** 10.1186/1471-2407-8-333

**Published:** 2008-11-13

**Authors:** JianTao Wu, Jun Gong, Juan Geng, YinXue Song

**Affiliations:** 1 Department of Gastroenterology, the Second Affiliated Hospital, Xi'an Jiaotong University School of Medicine, Xi'an, Shaanxi, 710004, PR China

## Abstract

**Background:**

Mucin alterations are a common feature of esophageal neoplasia, and alterations in MUC2 mucin have been associated with tumor progression in the esophagus. Bile acids have been linked to esophageal adenocarcinoma and mucin secretion, but their effects on mucin gene expression in human esophageal adenocarcinoma cells is unknown.

**Methods:**

Human esophageal adenocarcinoma cells were treated 18 hours with 50–300 μM deoxycholic acid, chenodeoxycholic acid, or taurocholic acid. MUC2 transcription was assayed using a MUC2 promoter reporter luciferase construct and MUC2 protein was assayed by Western blot analysis. Transcription Nuclear factor-κB activity was measured using a Nuclear factor-κB reporter construct and confirmed by Western blot analysis for Nuclear factor-κB p65.

**Results:**

MUC2 transcription and MUC2 protein expression were increased four to five fold by bile acids in a time and dose-dependent manner with no effect on cell viability. Nuclear factor-κB activity was also increased. Treatment with the putative chemopreventive agent aspirin, which decreased Nuclear factor-κB activity, also decreased MUC2 transcription. Nuclear factor-κB p65 siRNA decreased MUC2 transcription, confirming the significance of Nuclear factor-κB in MUC2 induction by deoxycholic acid. Calphostin C, a specific inhibitor of protein kinase C (PKC), greatly decreased bile acid induced MUC2 transcription and Nuclear factor-κB activity, whereas inhibitors of MAP kinase had no effect.

**Conclusion:**

Deoxycholic acid induced MUC2 overexpression in human esophageal adenocarcinoma cells by activation of Nuclear factor-κB transcription through a process involving PKC-dependent but not PKA, independent of activation of MAP kinase.

## Background

**C**hanges in the characteristics of the surface epithelial mucins is the hallmark of Barrett's metaplasia, dysplasia and adenocarcinoma of esophagus[1,2]. MUC2, a high molecular weight glycoprotein, is the major secreted mucin in the large and small intestine[3,4]. Human esophageal adenocarcinoma and cell lines derived from tumors can differ significantly in the amount of MUC2 mucin synthesized and these differences correlate with altered biochemical and biologic properties including those with relevance to invasion and metastasis, MUC2 is expressed in esophageal carcinoma cell lines, and patients with esophageal carcinomas characteristically present with advanced-stage disease[1,5,6].

Bile acids, fractions of duodenogastricoesophageal reflux (DGER) have been detected in patients with extensive esophageal mucosal damage, have been reported to promote esophageal carcinogenesis[7,8]. These bile acids, primarily deoxycholic acid (DCA), are cytotoxic to esophageal cells[8], and are established tumor promoters in animal models[9]. In esophageal adenocarcinoma, DCA is believed to contribute to carcinogenesis during reflux where reluxates enter the lower esophagus[10]. Bile acids also have been reported to stimulate invasion and metastasis of esophageal carcinoma cells via activation of multiple signaling pathways [11-13]. Although regulations of MUC1 and MUC4 mucin genes by bile acids, such as DCA, CDCA and TCA, in human oesophageal cancer cells have been the thorough extensive study [14-16], the mechanisms responsible for regulation of MUC2 expression in the esophageal adenocarcinoma cells remain unknown.

In the current study, we sought to determine the effects of bile acids on MUC2 gene expression in esophageal adenocarcinoma cells and the molecular mechanisms involved. We find that bile acids induce MUC2 expression in human esophageal adenocarcinoma cells at the level of transcription through a process that involves protein kinase C (PKC)-dependent activation of Nuclear factor-κB (NF-κB), primarily a MAP kinase-independent.

## Methods

### Materials

Deoxycholic acid (DCA), chenodeoxycholic acid (CDCA), and taurocholic acid (TCA) were obtained from Sigma (St. Louis, USA). CAPE, Calphostin C, U0126 (1,4-diamino-2, 3-dicyano-1,4-bis(2-aminophenylthio)butadiene), PD98059 (2'-amino-3'-methoxyflavone), and H-8 (PKA inhibitor) (N-[2-(methylamino)ethyl]-5-isoquinolinesulfonamide dihydrochloride) were purchased from Calbiochem (San Diego, CA). Mouse monoclonal antibody (MoAb) CCP-58, specific for MUC2 glycoprotein, was obtained from Novocastra (Newcastle, UK). Antibodies for Nuclear factor-κB (NF-κB) p65, extracellular signal-regulated kinase (ERK1/2), JNK, P38 and phospho-ERK1/2, JNK, P38 were obtained from Cell Signaling Technology (Beverly, MA), aspirin, secondary antibodies and anti-beta-actin MoAb was obtained from Sigma (USA). FuGENE 6 transfection reagent was from Roche (Indianapolis, IN).

### Cell Culture and Treatment

SEG-1 is a BE adenocarcinoma cell line, the cell line were cultured in Dulbecco's Modified Eagle Medium (Invitrogen, Carlsbad, Calif) supplemented with 10% heat-inactivated fetal bovine serum (Invitrogen) and 100 U/mL penicillin G and 100 μg/mL streptomycin (Invitrogen) at 37°C in a humidified incubator containing 5% carbon dioxide.

SEG-1 cells were plated in regular medium for 36 hours. The medium was then replaced with 0.5% FBS for an additional 24 hours. Cultures were then treated with bile acids. For inhibitor assays, SEG-1 cells were pretreated with inhibitors for 1 hour before exposure to DCA for an additional 18 hours. Calphostin C was used under a fluorescent lamp of 13 W located 15 cm above the plates.

For determining the effects of bile acids on viability, cells were treated for 24 hours with ≤ 200 μM DCA, CDCA, or TCA, then detected with The CellTiter-Fluor™ Assay kit(Promega BioSciences, San Luis Obispo, CA), according to the manufacturer's protocol

### Protein Extraction and Western Blotting

Cellular proteins from treated SEG-1 cells were prepared in 40 mM Tris-HCI, pH 6.9, 150 mM NaCl, 2 mM ethylenediaminetetraacetic acid, 100 mM sodium fluoride, 10 mM sodium pyrophosphate, 2 mM orthovanadate, 1% Triton X-100, 1% Nonidert P40 (NP-40), 0.3 mM phenylmethanesulfonyl fluoride, and 1 mini tablet protein inhibitor (sigma, USA). Separate cytosol and nuclear protein lysates were prepared by using the Active Motif Nuclear Extract Kit (Active Motif Europe, Rixensart, Belgium), according to the manufacturer's protocol. For routine quantitation of proteins, following the manufacturer's protocol (Pierce, Rockford, IL). Equal amounts of protein samples were subjected to sodium dodecyl sulfate-polyacrylamide gel electrophoresis on either 3–8% Tris-acetate gradient gels for MUC2 detection or 10% Tris-glycine gels for detection of other proteins. After gel electrophoresis and transfer to nitrocellulose, the membranes were stained in 0.5% Ponceau S with 1% acetic acid to confirm the equal loading and transfer efficiency. Membranes were incubated at 4°C overnight in a blocking solution containing 5% bovine skim milk and 0.1% Tween 20 (Fischer Scientific, Pittsburgh, PA) in TBS (10 mM Tris-HCl with 150 mM NaCl, pH 7.6), then probed with specific primary and secondary antibodies conjugated to horseradish peroxidase. Immunoreactive bands were visualized by chemiluminescence solution and exposure to X-ray film.

### RNA Isolation and RT-PCR

Total RNA was isolated using TriReagent (Molecular Research Center Inc.), and 3 μg was primed with random hexamers and reverse transcribed using Superscript II (Invitrogen) in a final volume of 50 μl. One microliter of this mixture was PCR-amplified in a 10 μl reaction using AmpliTaq DNA polymerase (Applied Biosystems) with the addition of 5% dimethyl sulfoxide. Primers for *MUC2 *were (forward) 5'-TGC CTG GCC CTG TCT TTG-3' and (reverse) 5'-CAG CTC CAG CAT GAG TGC-3'; *NF-κB p65 *were (forward) 5'-GCG AGA GGA GCA CAG ATA CC-3' and (reverse) 5'-CTG ATA GCC TGC TCC AGG TC-3'. The PCR reaction mixture was denatured at 95°C for 5 min followed by 30 cycles at 93°C for 30 s, 60°C for 30 s, and 72°C for 30 s. Alternatively, blocked and unblocked primers for beta-actin (forward) 5'-ATC TGG CAC CAC ACC TTC TAC AAT GAG CTG C-3'; (reverse) 5'-CGT CAT ACT CCT GCT TGC TGA TCC ACA TCT G-3' were used to amplify this message as an internal control. All the primers were synthesized by Sangon (Shanghai, China). All PCR products were analysed by Gel-Pro analyser version 3.1 software.

### Transient Transfection and Luciferase Reporter Assays

Plasmids were prepared using the Genopure plasmid midi kit from Roche (Indianapolis, IN). Methods to measure the *MUC2 *promoter activity with luciferase as a reporter have been reported previously [17-19]. Upstream fragments of 2665 base pair (bp) from immediately adjacent to the 5' translation start site of human *MUC2 *[GenBank accession number U67167] [17] were generated by routine polymerase chain reaction from human genomic DNA, using the following primer pairs (forward) 5'-GAGGCTAGCCCGGGCTTCCTGGTGAGTC-3', and (reverse) 5'-GAGCTCGAGCATGGTGGCTGGCAGGGGC-3'. The 2665-bp fragment was then inserted upstream of the luciferase reporter in the pGL3-basic vector, according to the instructions provided by the manufacturer (Promega). DNA sequencing was performed to verify the correct clone.

NF-κB transient transfection assays have been reported previously[20,21]. The cells were transfected using the SuperFect reagent (Qiagen) with a *NF-kB *luciferase reporter (Clontech) plasmid and a SV40 promoter-driven beta-galactosidase expression plasmid to normalize the transfection efficiency. After additional 24 h, the cells were harvested in phosphate-buffered saline, lysed in luciferase lysis buffer (Promega), and assayed for luciferase and beta-galactosidase activities using the Promega luciferase assay system (Promega).

For transient transfection, SEG-1 cells were seeded at a concentration of 6 × 10^5 ^cells per well in 6-well plates. After overnight, the cells in each well were transfected with DNA (3 μg of *MUC2 *or *NF-κB*-luciferase reporter plasmid and 0.2 μg of pCH110) by using 3 μL of FuGENE6 (Roche) by following the manufacturer's protocol. After a 24-hour exposure to the transfection mixture, the cells were incubated in medium containing 10% FBS and different concentrations of bile acids or inhibitors for an additional 18 hours and then harvested for measurement of beta-galactosidase activity and luciferase activity. The latter was measured by using the Promega luciferase assay system according to the manufacturer's protocol using a TriStar LB941 (Berthold; Germany). Luciferase was normalized to the beta-galactosidase activity to account for differences in transfection efficiency.

### NF-κB p65 siRNA Transfection

SignalSilence NF-κB p65 siRNA was purchased from Cell Signaling Technology (Danvers, MA). siCONTROL nontargeting siRNA purchased from Dharmacon (Chicago, IL) was used as a control siRNA. Cells were transfected with siRNA using Oligofectamine transfection reagent (Invitrogen) for 72 h according to the manufacturer's instructions. To confirm the efficacy of NF-κB p65 siRNA, NF-κB p65 mRNA and protein were analyzed using RT-PCR and Western Blotting. The cytotoxic effect of NF-κB p65 siRNA on SEG-1 cells was determined by CellTiter-Fluor™ Assay (Promega, Madison, WI) according to the manufacturer's protocol.

### Statistical Analyses

Results were expressed as means ± SD, and statistical significance was determined using by ANOVA. Differences of P < 0.05 were considered significant. All experiments were performed in triplicate.

## Results

### Effects of Bile Acids on MUC2 Expression and Transcription

**Figure 1:**
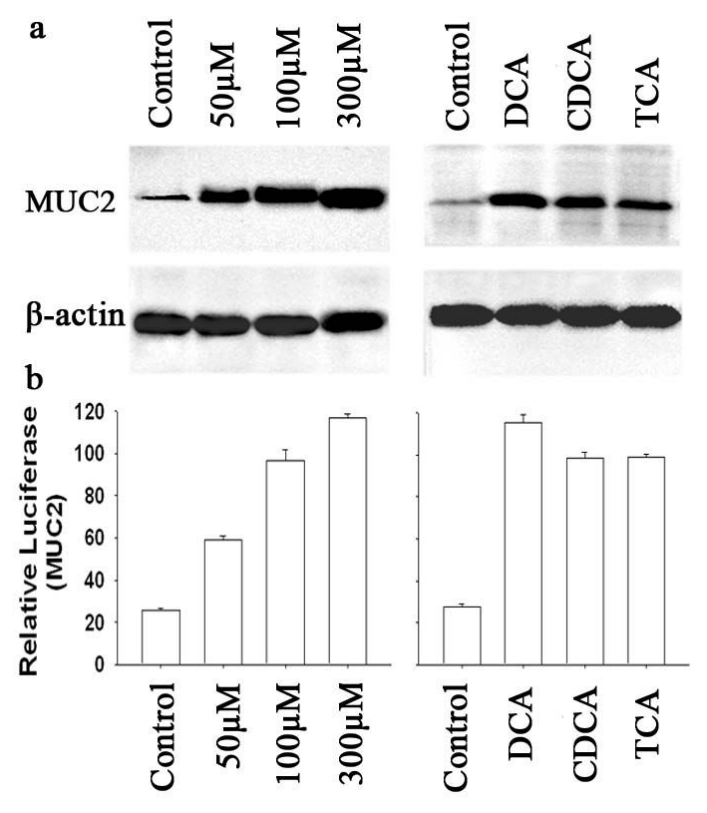
**Effect of bile acids on MUC2 expression.** (a) SEG-1 cells were treated for 18 hours with 50–300 μM DCA (left) or with 100 μM DCA, CDCA, or TCA (right). Total cellular protein was isolated and subjected to Western blotting for MUC2 and β-actin. (b) SEG-1 cells were transfected with MUC2 promoter luciferase construct, then treated for 18 hours with 50–300 μM DCA (left) or with 100 μM DCA, CDCA, or TCA (right). Luciferase activity for the MUC2 reporter was measured and normalized to beta-galactosidase activity. Values shown represent the means ± SD of triplicate experiments.

SEG-1 treated with DCA, CDCA, and TCA. Viability of SEG-1 cells was not significantly affected by treatment for 24 hours with 200 μM DCA (data not shown). The addition of DCA in 50–300 μM for 18 hours to SEG-1 increased MUC2 protein expression in a dose-dependent manner with a maximum increase of approximately 4-fold (Fig. 1a). Different bile acids may have different biologic effects[22,23]. To examine the effects of different bile acids on MUC2 induction, we treated SEG-1 with DCA, CDCA, and TCA for 18 hours at a concentration of 100 μM. As shown in Figure 1a, all three bile acids increased MUC2 protein expression, with DCA having the strongest effect.

To determine whether bile acids increase MUC2 promoter activity, we employed an MUC2 promoter luciferase construct, which contains a 2205-bp human MUC2 5'-flanking region fused to a luciferase reporter gene. After transient transfection, cells were treated with different concentrations of bile acid and luciferase activities were determined (Fig. 1b). DCA induced MUC2 promoter-driven luciferase activities in a dose-dependent manner in SEG-1 cells, with a maximum 4–5 fold increase with 300 μM DCA. All tested bile acids induced MUC2 transcription activity to a varying extent at a concentration of 100 μM for 18 hours.

### Effects of Bile Acids on NF-κB Expression and Transcription

**Figure 2:**
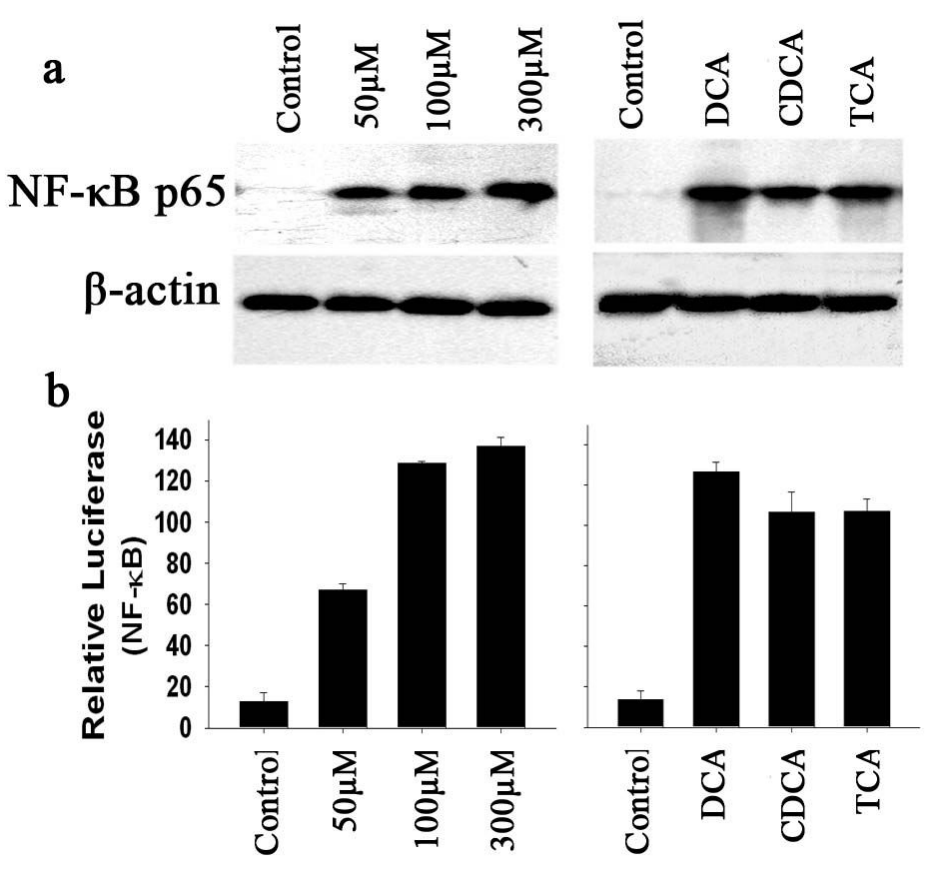
**Effect of bile acids on Nuclear factor-κB (NF-κB) expression.** (a) SEG-1 cells were treated for 18 hours with 50–300 μM DCA (left) or with 100 μM DCA, CDCA, or TCA (right). Cellular nuclear protein was subjected to Western blotting for NF-κB p65 and β-actin. (b) SEG-1 cells were transfected with the NF-κB luciferase reporter plasmid, incubated for 24 hours, then treated for an additional 18 hours with 50 – 300 μM DCA (left) or with 100 μM DCA, CDCA, or TCA (right). Luciferase activity for NF-κB reporter plasmid was measured and normalized to beta-galactosidase activity. Values shown represent the means ± SD of triplicate experiments.

We evaluated NF-κB p65 protein levels and gene transcription in the esophageal adenocarcinoma cell line. SEG-1 treated with DCA in 50–300 μM for 18 hours to SEG-1 increased NF-κB p65 protein expression in a dose dependent fashion with a maximum increase of approximately 5-fold (Fig. 2a).

To examine the effects of different bile acids on NF-κB induction, we treated SEG-1 with DCA, CDCA, and TCA for 18 hours at a concentration of 100 μM. As shown in Figure 2a, all three bile acids increased NF-κB p65 protein expression, with DCA having the strongest effect.

To determine whether bile acids increase NF-κB gene transcription, we employed a NF-κB promoter luciferase construct, luciferase reporter gene driven by the NF-κB promoter, was transiently transfected into SEG-1. After 24 hours, the transfected cells were treated with increasing doses of DCA and with different bile acids in 100 μM for 18 hours (Fig. 2b). DCA induced NF-κB promoter-driven luciferase activities in a dose dependent manner in SEG-1 cells, with a maximum 5–6 fold increase at a concentration of 300 μM. All tested bile acids induced NF-κB luciferase activities to a varying extent at a concentration of 100 μM for 18 hours, with DCA having the strongest effect (Fig. 2b), a result consistent with induction of MUC2 expression (Fig. 1b).

### Requirement of NF-κB for Induction of MUC2 by DCA

**Figure 3:**
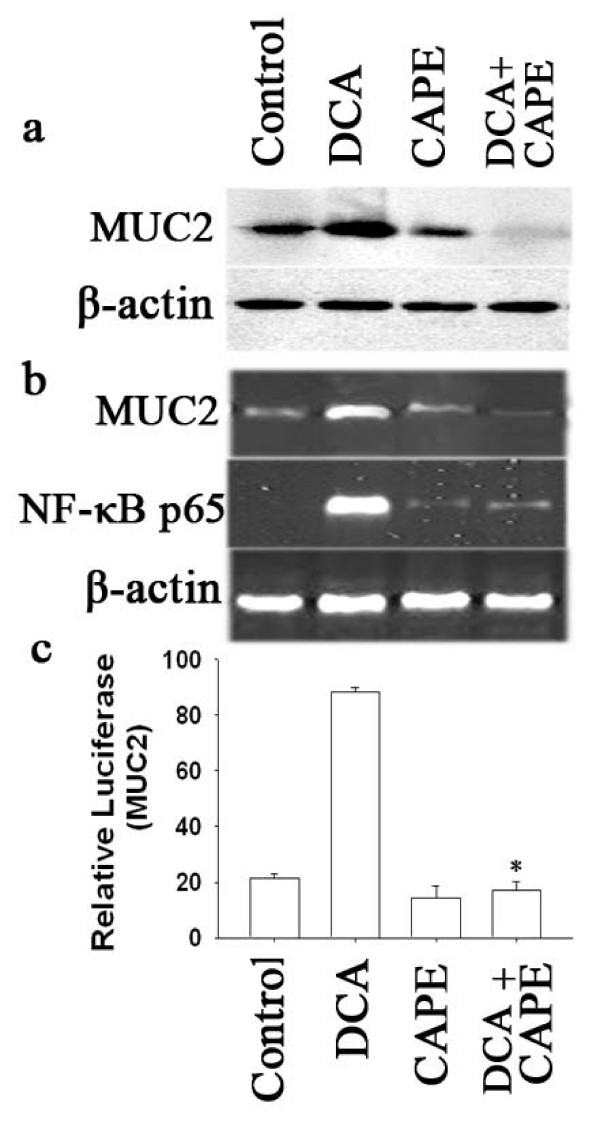
**Effect of CAPE on expression of MUC2.** SEG-1 cells were pretreated with or without 10 μg/ml CAPE for 1 h and then treated for 18 hours with or without 100 μM DCA. (a) Total cellular protein was subjected to Western blotting for MUC2 and β-actin. (b) The isolated RNA samples were analyzed by RT-PCR for MUC2, NF-κB p65 and β-actin. (c) SEG-1 cells were transfected with MUC2 promoter luciferase construct, then treated for 18 hours with or without 100 μM DCA in the presence or absence of CAPE. Luciferase activity for MUC2 was measured and normalized to beta-galactosidase activity. (means ± SD of triplicate assays, * *p *< 0.05, for inhibition compared with assays without added inhibitor).

Previous reports have suggested that NF-κB is important in mediating bile acids signaling[8,24]. We therefore assessed the effect of NF-κB on expression of MUC2 induced by DCA in SEG-1 esophageal adenocarcinoma cells. CAPE, an inhibitor of NF-κB activation, completely blocked the induction of MUC2 by DCA (Fig. 3). This result was confirmed using Western Blotting to estimate changes in protein levels (Fig. 3a) and RT-PCR in MUC2 and NF-κB p65 transcript levels (Fig. 3b). To determine whether CAPE blocked MUC2 promoter activity, we employed an MUC2 promoter luciferase construct, which contains a 2205-bp human MUC2 5'-flanking region fused to a luciferase reporter gene. After transient transfection, cells were treated with 100 μM DCA for 18 hours and luciferase activities were determined (Fig. 3c). CAPE inhibite MUC2 promoter-driven luciferase activities in SEG-1 cells treated with DCA.

**Figure 4:**
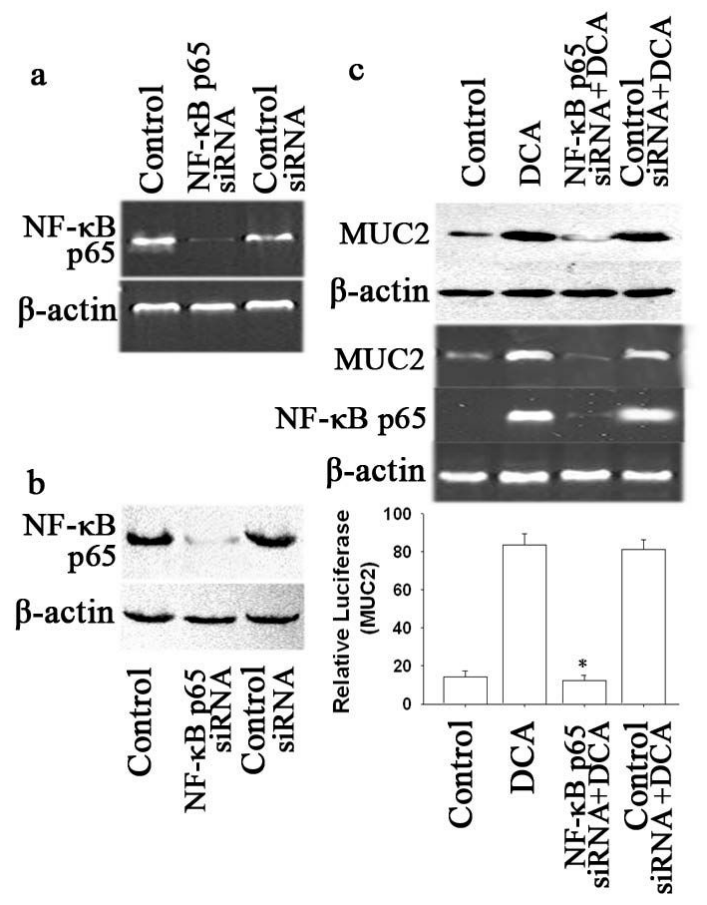
**Effects of NF-κB p65 siRNA on expression of MUC2.** SEG-1 cells were transfected with Nuclear factor-κB (NF-κB) p65 siRNA, and its effect on NF-κB p65 mRNA was analyzed using (a) RT-PCR, and (b) Western blotting. (c) Cells were treated with 100 μM DCA for 18 hours after exposure to siRNA targeting NF-κB p65, (Upper panel) SEG-1 cells lysates were analyzed in Western blotting for MUC2 and β-actin, (Middle panel) The isolated RNA samples were analyzed by RT-PCR for MUC2, NF-κB p65 and β-actin, (Lower panel) SEG-1 cells were transfected with human MUC2 promoter luciferase construct, then treated for 18 hours with or without 100 μM DCA after with or without exposure to siRNA targeting NF-κB p65. Luciferase activity for MUC2 was measured and normalized to beta-galactosidase activity. (means ± SD of triplicate assays, * *p *< 0.05, for NF-κB p65 siRNA compared with assays without NF-κB p65 siRNA).

Whether NF-κB p65 involvement in DCA induced MUC2 activation, it was tested by transfection of NF-κB p65-specific siRNA into SEG-1 cells. Specific knockdown of endogenous NF-κB p65 mRNA levels was observed (Fig. 4a, b), and the cells treated with NF-κB p65 siRNA also showed potent suppression of DC-induced MUC2 activation detected with in MUC2 transcript levels and Western Blotting in protein levels (Fig. 4c). To determine whether NF-κB p65 siRNA blocked MUC2 gene transcription, we employed an MUC2 promoter luciferase construct. In NF-κB p65 siRNA SEG-1 cells, after transient transfection MUC2 promoter luciferase reporter, cells were treated with 100 μM DCA for 18 hours and luciferase activities were determined (Fig. 4c), NF-κB p65 siRNA inhibited MUC2 promoter-driven luciferase activities in SEG-1 cells treated with DCA. These results suggest that the activity of NF-κB p65 involved in DCA-induced activation of MUC2 in SEG-1 cells.

**Figure 5:**
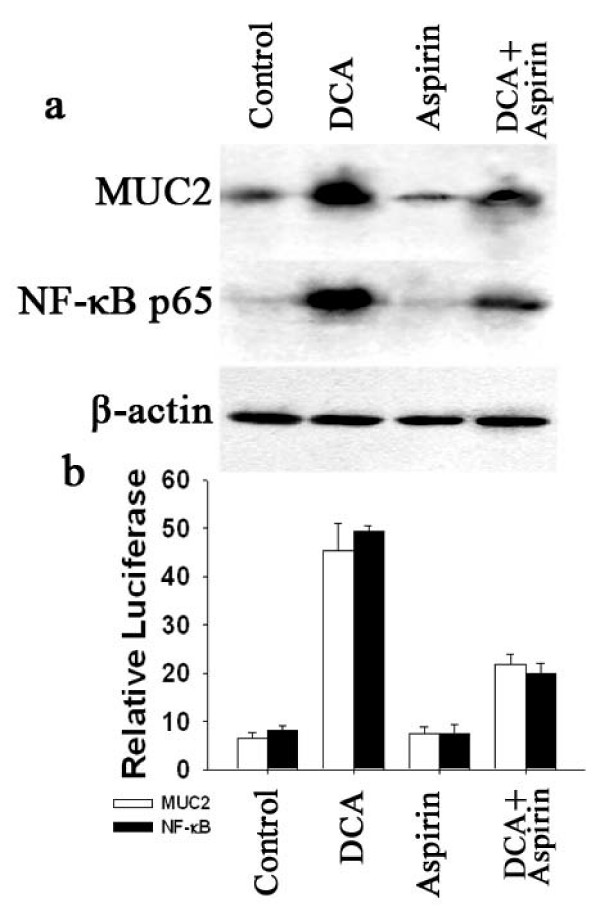
**Effect of aspirin on MUC2 and Nuclear factor-κB (NF-κB) p65 expression.** (a) SEG-1 cells were treated for 18 hours with or without 100 μM DCA in the presence or absence of 4 mmol/L aspirin. Total cellular and nuclear protein were subjected to Western blotting for MUC2, NF-κB p65, and β-actin. (b) SEG-1 cells were transfected with human MUC2 promoter luciferase construct or NF-κB luciferase reporter plasmid, then treated for 18 hours with or without 100 μM DCA in the presence or absence of 4 mmol/L aspirin. Luciferase activity for MUC2 or NF-κB was measured and normalized to beta-galactosidase activity. Values shown represent the means ± SD of triplicate experiments.

Although the precise mechanism by which aspirin inhibits esophageal tumorigenesis remains to be elucidated, it has been reported to inhibits NF-κB nuclear translocation in the esophageal squamous cell carcinoma cell line[25]. We examined the effect of aspirin, a potential chemopreventive agent in the prevention or treatment of esophageal cancer, on expression of MUC2 induced by DCA. In SEG-1 esophageal adenocarcinoma cells treated with DCA (100 μM) for 18 hours, the basal levels of MUC2 and NF-κB p65 proteins were decreased by treatment with 4 mmol/L aspirin, there was also partial inhibition of the bile acid-dependent induction of MUC2 and NF-κB p65 (Fig. 5a). In assays of MUC2 transcription and NF-κB transcriptional activity, aspirin also had effect on basal activity, and inhibited the bile acid-dependent induction of MUC2 and NF-κB activity (Fig. 5b).

### Involvement of Protein Kinase C but Not Protein Kinase A in Induction of MUC2 by DCA

**Figure 6:**
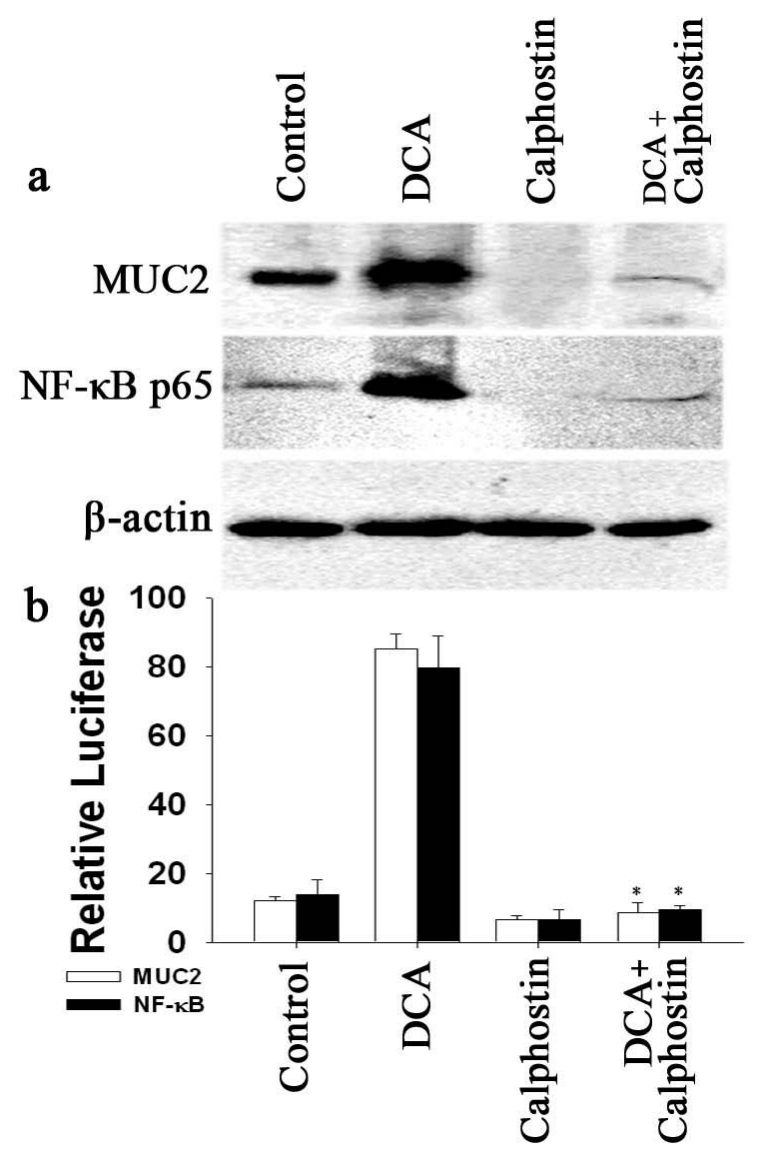
**Effect of calphostin C on MUC2 and NF-κB p65 expression.** SEG-1 cells were treated for 18 hours with or without 100 μM DCA in the presence or absence of 5 nM calphostin C. (a) Total cellular and nuclear protein were subjected to Western blotting for MUC2, NF-κB p65, and β-actin. (b) SEG-1 cells were transfected with MUC2 promoter Luciferase construct or NF-κB Luciferase reporter plasmid, then treated for 18 hours with or without 100 μM DCA in the presence or absence of 5 nM calphostin C. Luciferase activity for MUC2 or NF-κB was measured and normalized to beta-galactosidase activity. (means ± SD of triplicate assays, * *p *< 0.05, for inhibition compared with assays without added inhibitor).

It has been reported that bile acids activate PKC in esophageal adenocarcinoma cells[10,26]. Previous results have also shown that the PKC inducer PMA and bile acids increase MUC2 expression in colon carcinoma cell line, and that this is prevented by the PKC inhibitor, calphostin C[27,28]. However, it is unclear whether induction of MUC2 by bile acids is mediated by PKC in esophageal adenocarcinoma cells. When SEG-1 cells were treated with 6 nM calphostin C, there was almost complete inhibition of basal and bile acid-induced MUC2 protein and NF-κB p65 protein expression (Fig. 6a). This was accompanied by suppression of MUC2 transcription and of NF-κB transcriptional activity (Fig. 6b). These results indicate that PKC is involved in the NF-κB dependent induction of MUC2 expression by DCA.

**Figure 7:**
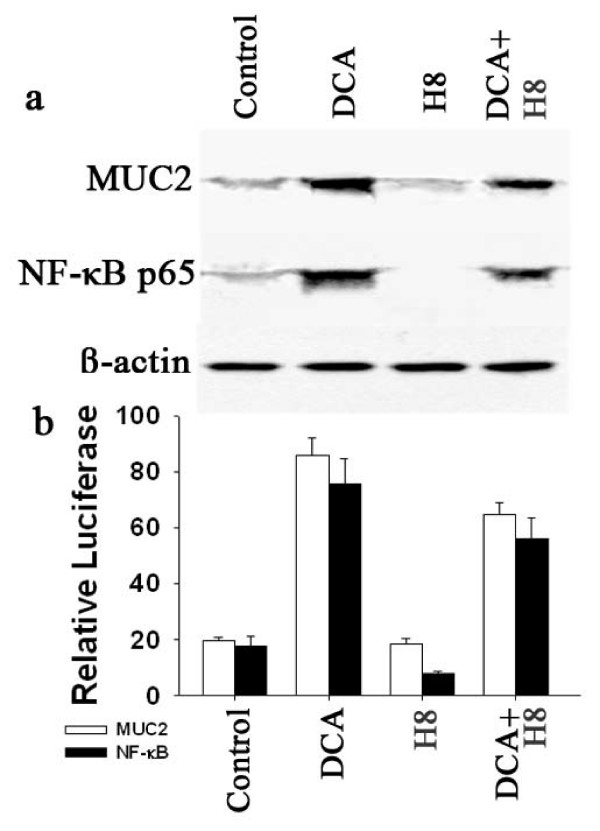
**Effect of H-8 on MUC2 and NF-Kb p65 expression. **(a) SEG-1 cells were treated for 18 hours with or without 100 μM DCA in the presence or absence of 5 μM H-8. Total cellular protein and nuclear protein were subjected to Western blotting for MUC2, NF-κB p65, and β-actin. (b) SEG-1 cells were transfected with the MUC2 promoter luciferase construct or the NF-κB luciferase reporter plasmid, then treated for 18 hours with or without 100 μM DCA in the presence or absence of 5 μM H-8. Luciferase activity for MUC2 or NF-κB was measured and normalized to beta-galactosidase activity. Values shown represent the mean and standard error of the mean of triplicate experiments.

There has been reported that PKA is involved in bile acid induce MUC2 expression, and that this is prevented by the PKA inhibitor, H-8[28]. We also examined the effect on MUC2 induction by DCA of specific protein kinase inhibitor, H-8 (N-[2-(methylamino)ethyl]-5-isoquinolinesulfonamide dihydrochloride). In our study, however, H-8 did not inhibited levels of MUC2 protein and NF-κB p65 protein both in the presence and absence of DCA in SEG-1 cells (Fig. 7a). There was also not a decrease in MUC2 transcription and in NF-κB transcriptional activity (Fig. 7b). These results suggest that the DCA-dependent induction of MUC2 depends on PKC but not PKA.

### Induction of MUC2 by DCA is not dependent of MAPK

**Figure 8:**
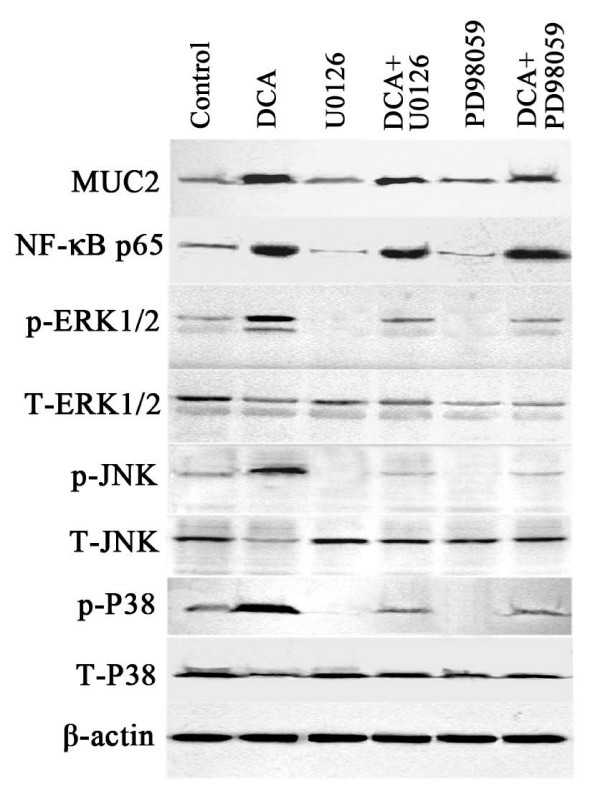
**Effect of U0126 and PD98059 on MUC2 and NF-κB p65 expression.** SEG-1 cells were treated for 18 hours with or without 100 μM DCA in the presence or absence of 8 μM U0126 and 60 μM PD98059. Total cellular and nuclear protein were subjected to Western blotting for MUC2, NF-κB p65, phosphorylation of ERK1/2, JNK, P-38, and total ERK1/2, JNK, P-38 and β-actin.

A previous study found that specific inhibitors of MAP kinase, U0126 and PD98059, inhibited the induction of MUC2 by bile acids[28]. To investigate the role of MAPK in the induction of MUC2 and NF-κB by DCA in esophageal adenocarcinoma cells, we examined phosphorylation of ERK1/2, JNK, P38 kinases and total ERK1/2, JNK, P38 kinases after treatment of SEG-1 cells with 100 μM DCA for 18 hours. The DCA had effect on the levels of phosphorylated ERK1/2, JNK, and P-38 or total ERK1/2, JNK, and P-38 kinases (Fig. 8). To further investigate the effection of MAPK on the induction of MUC2 and NF-κB, we used U0126 and PD98059 to selectively block the activity of MAPK, we found that these inhibitors did not block the DCA-dependent increase in MUC2 protein and NF-κB protein, although both U0126 and PD98059 did suppress phosphorylation of ERK1/2, JNK, and P38. These results suggest that expression of MUC2 and NF-κB treated with DCA is not dependent on MAPK in SEG-1.

## Discussion

The effects of bile acids on mucin gene expression in esophageal adenocarcinoma cells have not been well studied. Bile acids has been reported to increase the secretion of MUC2 in esophageal cells[29], but MUC2 gene expression and the molecular events responsible for MUC2 gene expression were not studied in esophageal adenocarcinoma cells. In the current study, we find that bile acids increase MUC2 expression in SEG-1 esophageal adenocarcinoma cells, and the transcriptional activity of MUC2 promoter reporter construct transiently transfected into SEG-1 was increased by DCA and other bile acids in a dose-dependent fashion, indicating that bile acid-induced MUC2 up-regulation occurs at the transcriptional level.

NF-κB is an important transcription factor that mediates expression of multiple genes in important biologic processes including cell growth, apoptosis, and transformation [30-32]. We postulated that NF-κB could play a role in the induction of MUC2 by bile acids. Our data indicate that CAPE, an inhibitor of NF-κB translocation, it reduced endogenous as well as bile acid up-regulated MUC2 transcription, in addition NF-κB expression and transcription activity coincided with MUC2 induction, and inhibition of NF-κB expression and activity efficiently suppressed bile acid-mediated up-regulation of MUC2, indicating that NF-κB is involved in MUC2 transcription induced by bile acid. Furthermore, it is confirmed by NF-κB p65 siRNA can also blocked MUC2 expression induced by bile acid. Activation of NF-κB is mediated through phosphorylation, ubiquination, and subsequent degradation of inhibitor IκB, this enables the free NF-κB p65 to translocate to the nucleus and activate target genes[33]. Our study shows that NF-κB p65 expression can be induced by DCA, suggesting degradation of inhibitor IκB may be involved in this pathway. Previous studies have indicated that NF-κB is involved in expression of MUC2[34,35], as the transcriptional competence of the NF-κB cis element was demonstrated containing the same region of the MUC2 promoter from bases ~1528 to ~1307 [27], implicating MUC2 promoter may be activated via expression of NF-κB induced by bile acids. Other transcription factors that have been shown to regulate MUC2 expression in other contexts include SP-1, CDX-2, and GATA-5 [36-38]. It is likely that these transcription factors activities may be also required for MUC2 expression in esophageal adenocarcinoma cells, but this has not yet been established.

The transcriptional activity of NF-κB is also enhanced directly by phosphorylation at various sites on its subunits. The kinases responsible for these phosphorylations may include PKA, and PKC. Activation of PKC by bile acids is well documented, and may be one mechanism of bile acid induced carcinogenesis [10,26]. We found that the PKC inhibitor calphostin C strongly blocked NF-κB and MUC2 induction by DCA, indicating that PKC is involved in the bile acid-dependent induction of MUC2. PKA has been reported that it is involved in bile acids stimulate MUC2 expression, although the bile acid-dependent induction of MUC2 depends less on PKA than on PKC[28]. However, our data indicated that the protein kinase A inhibitor, H-8, is not effective in blocking bile acid dependent induction of MUC2 and NF-κB, suggesting expression of MUC2 induced by DCA independent on PKA. In contrast to previous work on MUC2 induction by PMA via the ERK cascade, we find that the induction of MUC2 by bile acid is independent of MAP kinases in SEG-1 esophageal adenocarcinoma cells. Although treatment with DCA did affect the phosphorylation of ERK1/2, JNK, and P38 Kinase, U0126 and PD5089, inhibitors of MAP kinase, did not block MUC2 and NF-κB induction by DCA in SEG-1 cells, indicating MUC2 induction by DCA independent on the MAPK cascade.

## Conclusion

We conclude that treatment of human esophageal adenocarcinoma cells with DCA, up regulates MUC2 transcription by activation of NF-κB via PKC but not PKA, independent of MAP kinase. The biologic consequences of the induction of MUC2 expression by bile acids are unclear. Further studies are needed to confirm that whether induction of MUC2 by bile acids can increase the invasion potential of cells and their metastatic potential in vitro and in vivo. A more detailed understanding of the precise mechanisms by which bile acids induce MUC2 could also facilitate the development of chemopreventive strategies to diminish the risk of carcinogenesis and metastasis, particularly in esophageal adenocarcinoma.

## Competing interests

The authors declare that they have no competing interests.

## Authors' contributions

JTW and JGo designed the study. JTW carried out the molecular genetic studies, the sequence alignment, designed primers, western blots, siRNA transfections and analysis, performed the statistical analysis, drafted and revised the manuscript. JGe carried out the data analysis, drafted and corrected the manuscript and participated in the molecular genetic studies, in the sequence alignment, primer design and the statistical analysis. YXS corrected the drafts. All authors read and approved the final manuscript.

## Pre-publication history

The pre-publication history for this paper can be accessed here:


